# A computer vision approach for quantifying leaf shape of maize (*Zea mays* L.) and simulating its impact on light interception

**DOI:** 10.3389/fpls.2025.1521242

**Published:** 2025-06-23

**Authors:** Dina Otto, Sebastian Munz, Emir Memic, Jens Hartung, Simone Graeff-Hönninger

**Affiliations:** ^1^ Institute of Crop Science, Agronomy Department, University of Hohenheim, Stuttgart, Germany; ^2^ Department Sustainable Agriculture and Energy Systems, University of Applied Science, Freising, Germany

**Keywords:** leaf shape, leaf width, maize (*Zea mays* L.), computer vision, FSPM, light interception, simulations & learning

## Abstract

The precise determination of leaf shape is crucial for the quantification of morphological variations between individual leaf ranks and cultivars and simulating their impact on light interception in functional-structural plant models (FSPMs). Standard manual measurements on destructively collected leaves are time-intensive and prone to errors, particularly in maize (*Zea mays* L.), which has large, undulating leaves that are difficult to flatten. To overcome these limitations, this study presents a new camera method developed as an image-based computer vision approach method for maize leaf shape analysis. A field experiment was conducted with seven commonly used silage maize cultivars at the experimental station Heidfeldhof, University of Hohenheim, Germany, in 2022. To determine the dimensions of fully developed leaves per rank and cultivar, three destructive measurements were conducted until flowering. The new camera method employs a GoPro Hero8 Black camera, integrated within an LI-3100C Area Meter, to capture high-resolution videos (1920 × 1080 pixels, 60 fps). A semi-automated software facilitates object detection, contour extraction, and leaf width determination, including calibration for accuracy. Validation was performed using pixel-counting and contrast analysis, comparing results against standard manual measurements to assess accuracy and reliability. Leaf width functions were fitted to quantify leaf shape parameters. Statistical analysis comparing cultivars and leaf ranks identified significant differences in leaf shape parameters (p < 0.01) for term *alpha* and term *a*. Simulations within a FSPM demonstrated that variations in leaf shape can alter light interception by up to 7%, emphasizing the need for precise parameterization in crop growth models. The new camera method provides a basis for future studies investigating rank-dependent leaf shape effects, which can offer an accurate representation of the canopy in FSPMs and improve agricultural decision-making.

## 1 Introduction

Leaf shape is crucial for light interception and photosynthesis, directly influencing plant performance and developmental dynamics. It is characterized by variations in width along the blade. A precise assessment of leaf dimensions enables the quantification of morphological variability across different growth stages, leaf ranks, and cultivar-specific traits, fundamentally determining leaf area (Bos et al., 2000; Sanderson et al., 1981; Schrader et al., 2021).

Functional-structural plant models (FSPMs) integrate aspects of plant architecture, considering interactions between morphology and physiological processes such as photosynthesis. In FSPMs, plant organs, including leaves, are represented as individual entities extending sequentially in 3D from their point of origin. Although FSPMs are increasingly used, the simulations are still subject to significant uncertainties due to inaccurate input data, numerous model parameters and complex structures. With regard to the simulation of leaf shape and light interception, the development of robust data acquisition methods and precise mathematical descriptors is therefore crucial (Chapagain et al., 2022; Pasley et al., 2022).

Methodologies for assessing leaf shape typically involve destructive sampling and a manual procedure in which the leaf width is measured at multiple points along the leaf blade (Shi et al., 2019). However, this procedure is very time-consuming. Alternatively, the leaves can be pressed between 2 sheets of acrylic glass and photographed in order to digitally evaluate their length and width. For species such as maize (*Zea mays* L.) with large, undulating leaves, this method is particularly challenging and often leads to unreliable and inconsistent results (**Supplementary Figure 1**). In other studies, leaf shape was determined using image recognition software (Schrader et al., 2021) or integrating mathematical equations (Bos et al., 2000; Sanderson et al., 1981).

Dornbusch et al. (2011) introduced an empirical ‘leaf shape function’ using digital image analysis of destructively collected, flattened leaves from *Triticum aestivum*, *Hordeum vulgare*, and *Zea mays.* For maize leaves specifically, Sanderson et al. (1981) proposed a model to desribe the shape of fully developed leaves at any distance from the leaf tip. Based on a sine function, the model includes the term *alpha* (*alpha* > 0) to account for differences in leaf shape and term *a* (0.5 ≤ *a* ≤ 1) for the position of maximum leaf width. The model assumes an axis of symmetry for every leaf through the point where maximum leaf width occurs. Still, values are limited due to the fact that maximum width does not occur at the leaf base for maize leaves. Building on this equation (Bos et al., 2000), refined the model accounting for maximum width from the leaf base to the tip. While the model closely resembles the one proposed by Sanderson et al. (1981), it adds term *beta* (0 < *beta* < $\frac{\ln0.5\;}{\text{ln }beta}$ ) as a second leaf shape factor. For lower values of term *beta*, leaves tend to become wider towards the leaf tip relative to their maximum width. When *beta* = 1, the model simplifies to the mathematical model by Sanderson et al. (1981).

Although mathematical models provide useful approximations, in FSPMs predominantly a fixed function is used to describe leaves without considering rank- or cultivar-specific differences. Only a few studies have systematically examined maize leaf shape variability across ranks (Bos et al., 2000; Sanderson et al., 1981) or cultivars in relation to 3D modeling (Vos et al., 2010). Developing an efficient and accurate method for quantifying leaf shapes could enhance light interception modeling and improve cultivar selection. To address these challenges, our study aims to develop a novel, computer vision-based approach for precise leaf shape quantification. Thus, the objectives of this study were: (i) to develop a computer vision-based camera method to measure leaf width along the blade of destructively collected leaves, therewith providing a faster and more scalable alternative to the standard manual method measuring leaf width along the leaf blade; (ii) to compare the generated data by fitting the leaf width function, and determining leaf shape parameters; (iii) to investigate rank- and cultivar-specific differences in leaf shape; (iv) to test and quantify what influence these differences in leaf shape have on light interception within an FSPM.

## 2 Materials and methods

### 2.1 Field experiment

A field experiment with seven commonly used silage maize cultivars was conducted at the experimental station Heidfeldhof, University of Hohenheim (48°42’52”N 9°11’30”E, 401 m a.s.l.) in the growing season 2022. According to the World Reference Base for Soil Resources, the soil was classified as a loess-derived stagnic Luvisol with a pH of 7.2 and a humus content of 2% (Lehmkuhl et al., 2021). At the experimental site, the long-term average annual precipitation (1961–1990) was 679 mm with an average annual temperature of 8.7°C. In 2022, during the data collection period from April to August, the average precipitation amounted to 65.9 mm, and the average temperature was 15.9°C, compared to the long-term average of 74.7 mm and 14.8°C for the same time period (**Table 1**). In the field trial, seven commercially available, early-maturing silage maize cultivars – commonly cultivated under local growing conditions – were evaluated for differences in leaf shape: Amaroc, Benedictio, Figaro, LG30.258, Ricardinio, Ronaldinio, and Stabil (all from KWS Saat SE & Co. KGaA, Einbeck, Germany).

**Table 1:** Weather data observation period and long-term average at the experimental site Heidfeldhof, University of Hohenheim, Germany (48°42’52”N 9°11’30”E, 401 m a. s. l.).

Month	Precipitation 2022	Long-term precipitation (1961-1990)	Temperature 2022	Long-term temperature (1961-1990)
	∑	∑	Ø	Ø
	[mm]	[mm]	[°C]	[°C]
April	104.0	56.4	8.5	9.5
May	50.3	82.6	15.5	13.9
June	79.8	92.9	19.4	17.0
July	29.5	67.0	20.3	19.1

Six months before the sowing, the field was cultivated using a Claas Arion 640+ (CLAAS KGaA mbH, Harsewinkl, Germany), which featured a four-furrow plow (LEMKEN GmbH & Co. KG, Alpen, Germany). In the following spring, all plots were levelled to a depth of 0.20 m with a harrow (LEMKEN GmbH & Co. KG, Alpen, Germany). On 19 April 2022, the pre-emergence herbicide ‘Gardo Gold’ (Syngenta Crop Protection, Basel, Switzerland) was applied once at a rate of 4 L ha^-1^ with 400 L ha^-1^ water using a Fendt Farmer 275 S/SA (AGCO GmbH, Marktoberdorf, Germany) equipped with the spraying system Amazone UF 901 (Amazonen-Werke H. Dreyer SE & Co. KG, Hasbergen, Germany). On 21 April 2022, the field experiment was sown manually with a row width of 0.75 m and a density of nine plants m^-^². For fertilization, 80 kg N/ha AHL was applied on the 30th of May, 2022. The experimental set-up was a row-column design with four replicates and a total of 28 plots. The size of each plot was 2 × 2.25 m^2^.

#### 2.1.1 Destructive leaf sampling

To determine the dimensions of fully developed leaves for each rank per plant and cultivar, three destructive measurements (M1–M3) were carried out until flowering at 81 days after sowing (DAS): BBCH 14 (42 DAS; M1), BBCH 32 (64 DAS; M2), and BBCH 65 (81 DAS; M3). A single plant was sampled per plot and measurement date, and visible leaf collars were counted in ascending order with each leaf collar corresponding to a single leaf rank. Only fully developed leaves with visible collars were used for data collection. Senescent leaves were excluded, resulting in ranks 1 to 4 (M1), ranks 5 to 7 at (M2), and ranks 8 to maximum 16 or 17 (depending on the cultivar) (M3).

### 2.2 Leaf width measurement

#### 2.2.1 Manual method (I)

At each measurement date, maximum leaf length *L_max_* and maximum leaf width *W_max_* of each fully developed leaf per plant were measured manually with a ruler, similar to Bos et al. (2000). The measurements were taken from the leaf tip to the base. Depending on *L_max_*, each leaf was divided into evenly distributed segment points on the midrib, resulting in a minimum of six points and a maximum of eleven (M1 and M2) or twelve points (M3). Each segment point *i*, $W_{i}$ was then measured perpendicular to the midrib to follow the leaf contour systematically. At the first measurement point, leaf width was *W_1_* = 0 cm.

#### 2.2.2 Camera method (II) - a computer vision approach

**Figure 1:**
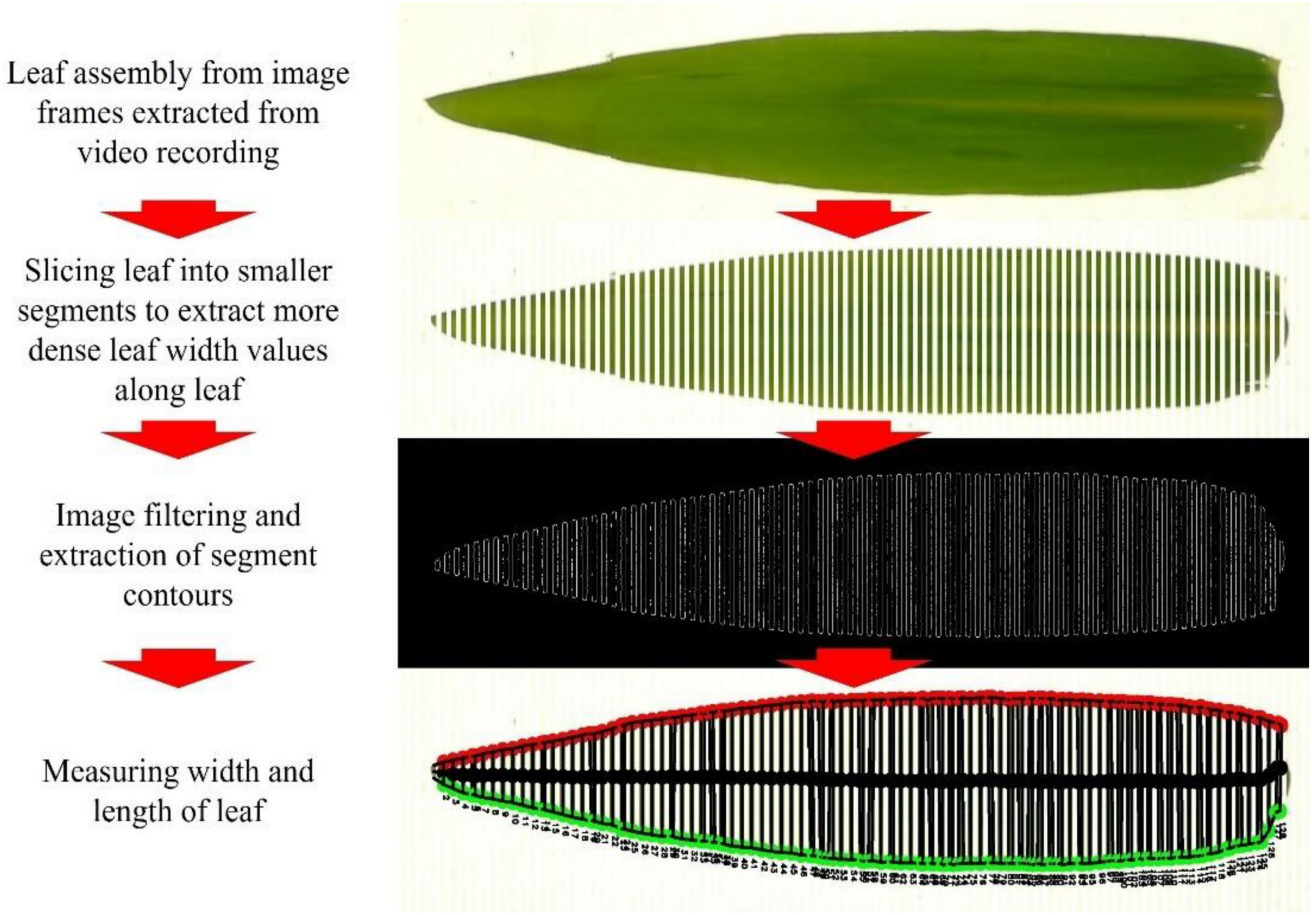
Complete schematic procedure of new camera method measurement approach for measuring leaf width and length.

The camera method (II) was developed as an image-based computer vision approach for determining the maximum leaf width of destructively collected leaves in a standard indoor measurement setting. This method integrates computer vision with the use of an LI-3100C Area Meter (LI-COR Environmental, Lincoln, NE, USA), which records the leaf area using a solid-state scanning camera. A mirror system reflects the objects under a fluorescent light source onto the camera in the rear housing. Therewith, measurements in a controlled shadow-free lighting environment should be guaranteed. By adding a camera for video recording to an LI-3100C Area Meter, leaf shape analysis can be performed ‘on the side’ in combination with other measurements such as leaf area estimation. The camera method (II) itself involves multiple steps, including leaf assembly from image frames, slicing the leaf image into smaller segments, image filtering and extraction of segment contours and determining leaf width (**Figure 1**). These steps will be explained in more detail in the following.

First, videos were recorded using a GoPro Hero8 Black camera (GoPro, San Mateo, CA, USA). The camera was embedded beneath the two transparent, motorized conveyor belts of the LI-3100C Area Meter, that transport leaves across a scanning platform, digitally accumulating total area of leaves analyzed in a session. Adjustable pressure rollers were used to flatten the curled maize leaves, ensuring precise surface quantification based on plant-specific characteristics. Individual maize leaves were placed on the lower transparent belt and processed through the system. Camera filter settings were optimized to enhance the contrast between the leaf and the background. Videos of each leaf, categorized by rank and plant, were recorded for 30 seconds at a resolution of 1920 × 1080 pixels, 60 frames per second, and saved in MP4 format. As the leaf moved across the scanning platform, the camera captured continuous video frames, which were extracted using the OpenCV library and assembled into full-sized leaf images. For scale reference, round greenish markers were placed on the LI-3100C Area Meter lamella during recording (**Supplementary Figure 2**). Image processing, including Gaussian blur and Canny edge detection, as well as analysis, were conducted using the cross-platform computer vision library OpenCV (Bradski, 2000). A semi-automated software solution for object detection and dimension measurement was developed in Python (van Rossum, 1995) incorporating a user interface built with QtDesigner (www.qt.io). The software, compiled into a Windows executable using PyInstaller (https://pyinstaller.org/en/stable/) is based on technical concepts from PyImageSearch (https://pyimagesearch.com) and is available as an open-source project on GitHub (https://github.com/memicemir/video_to_image_and_object_dimension_detection).

The algorithm first detects the coin-like reference object using contour detection, scanning from bottom to top. Based on the known width of this reference object (2.47 cm), a calibration metric is computed to determine real object dimensions as presented in (Equation 1):


(1) calibration metric=detected ‘coin' width (pixels)/known ‘coin' width (cm)

**Figure 2:**
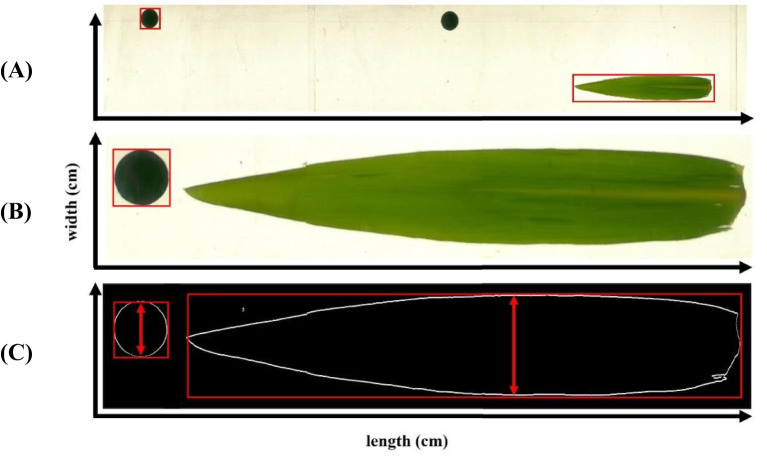
Image-based leaf dimensions (width and length) measurement procedure with reference (‘coin’ like) marker starting from image assembly from video recording **(A)**, referencing **(B)** to contour extraction **(C)**.

The Python program identifies the contours of detected objects in an image, using the first detected object – the coin-like reference – to establish a diameter calibration metric (**Figures 2A, B**). This metric defines the number of image pixels corresponding to a predefined real dimension (2.47 cm) and is essential for calculating the actual width of detected leaf objects (**Figure 2C**, red arrows).

**Figure 3:**
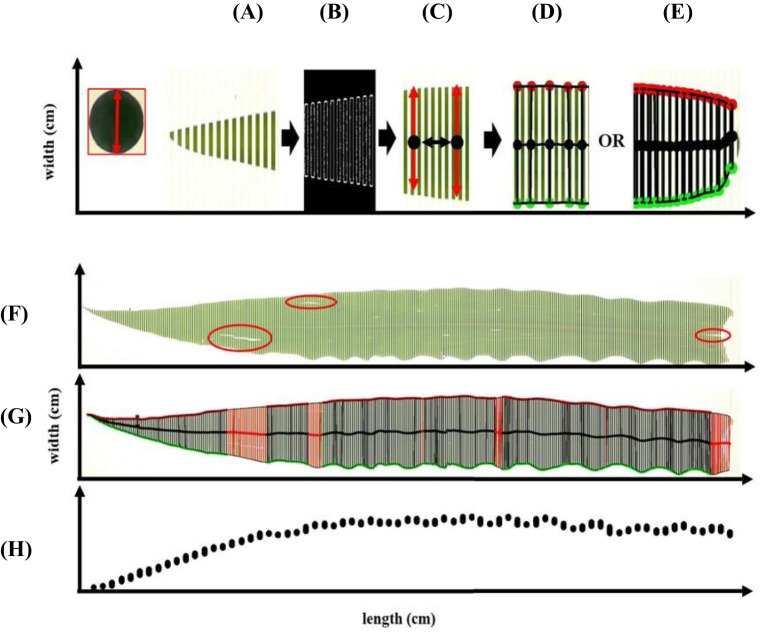
Leaf analysis (leaf width and length) of multiple segments in images **(A)** based on contour capture **(B)** to produce smoothed leaf segmented image **(C)** with optional measurement frequency **(D, E)**. Image analysis approach along irregular leaf shape and defects within the leaf **(F, G)** for producing (fitting) data points along the leaf **(H)**.

To enable frequent leaf width measurements along its entire length, the software automatically divides a full-size leaf into multiple segments (**Figures 3A-E**). The software automatically segments the leaf into multiple sections to enable frequent width measurements along its entire length, treating each segment as an individual object for precise dimension extraction. Image preprocessing includes Gaussian blur (**Figure 3A**) and Canny edge detection (**Figure 3B**) to produce a smoothed segmented image. The width of each segment is then calculated using the calibration metric (**Figure 3C**). Measurement frequency can be adjusted based on user preference and leaf morphology: less frequent measurements for regularly shaped leaves (**Figure 3D**) and more frequent leaf witdth sampling for irregularly shaped leaves to enhance accuracy along the leaf’s length (**Figure 3E**). The software detects only the outer edges. It ignores additional contours caused by fissures from pests or wind damage to ensure measurement accuracy (red areas in **Figure 3F, G**).

The final processing step resulted in a dense dataset of observed leaf width measurements for all designated leaf segments. The width of each segment was stored in an output file, allowing for the fitting of leaf width functions (**Figure 3H** **).** Validation was based on pixel-counting and green-non-green contrast analysis, which were converted to metric measurements. The image-derived leaf width data were compared to measurements obtained using the manual method (I). Data collection of the camera method in the scope of this study was conducted in 2022 at the Crop Science Institute, University of Hohenheim.

### 2.3 Fitting of leaf width function

The datasets from the three destructive measurements (M1, M2, M3) were merged into a comprehensive dataset. All ranks were checked graphically to ensure consistency across successive leaf positions. Leaf length and width values were normalized relative to their maximum measurement, allowing for standardized comparisons across leaves of different sizes. The leaf width (Equation 2) by Sanderson et al. (1981) was fitted to datasets of first, the manual method (I) and then, the camera method (II). Therewith, term *alpha* and term *a* were determined separately for each rank and cultivar and concerning the respective method (I) or (II). Model fitting was performed using the nlsLM package (R 4.1.0, R Core Team, 2021).

(Equation 2) by Sanderson et al. (1981):


(2) $$w_{i}=\text{sin}{\left(\frac{\pi }{2a}l_{i}\right)}^{\alpha }$$

where $w_{i}$ is the relative leaf width at relative length $l_{i}$ from the leaf tip, term *a* is the ‘ratio factor’ of $l_{i}$ with 0.5< *a* ≤ 1, at which maximum width occurs, term *alpha* is the ‘shape factor’ with α > 0. Estimates were obtained for the relative leaf width and term *alpha* and term *a.* Observed relative width values were regressed on estimated width values using manual method (I) and camera method (II). Adjusted R² and root mean square error (RMSE) were calculated. Regression analyses were conducted using SAS 9.4 (SAS Institute Inc., 2016). Model visualization was performed in R 4.1.0 (R Core Team, 2021).

### 2.4 Functional-structural plant model for simulating light interception

**Figure 4:**
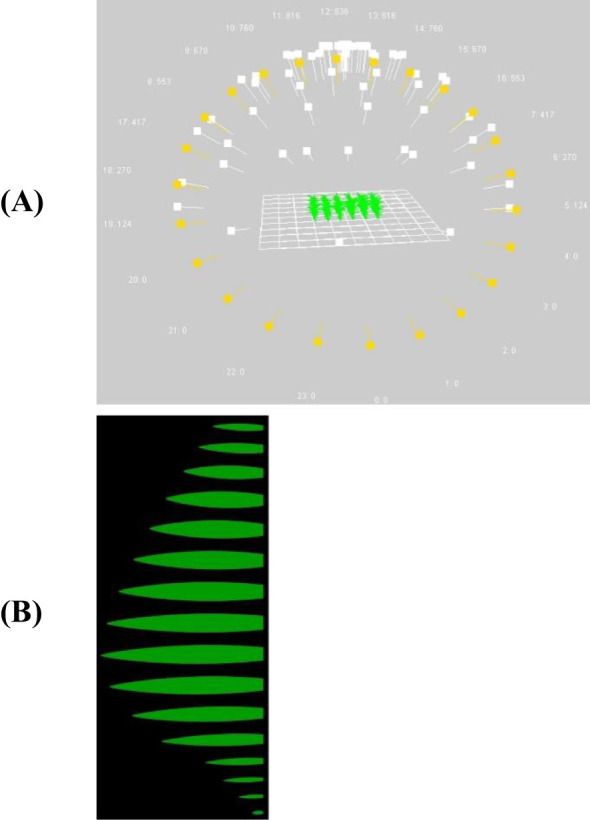
Visualization of the FSPM with 72 light sources in the scene (direct hourly light sources in yellow, diffuse light sources in white) **(A)** used to evaluate the effect of leaf shape on canopy light interception **(B)**.


For quantifying the effect of different leaf shapes on light interception, a functional-structural plant model (FSPM) was developed on the Growth Grammar-related Interactive Modeling Platform GroIMP (Kniemeyer, 2008). The primary goal of the simulations was to isolate the effect of leaf shape on light interception. The light distribution and interception of individual organs were simulated with the GPUFluxModel based on Monte-Carlo ray tracing (van Antwerpen, 2011). The light model was used with 200 M light rays and a reflection depth of 30. Simulations were performed with an increasing number of light rays and reflection depth until differences between multiple simulations of the same setup appeared negligible (< 0.001), based on observed mean values and standard deviations per treatment and simulation run. The hourly diurnal course of direct sunlight was calculated based on the latitude of the study site, and for the day of year 180 (**Figure 4A**). The fraction of diffuse sunlight was set to 0.2. The diffuse sunlight was simulated by 72 light sources arranged in six arrays across the hemisphere. The light rays were emitted from these light sources into the scene and reflected, transmitted, or absorbed by the leaves. Reflectance and transmittance were set to 0.0923 and 0.0127, respectively (Evers et al., 2010; Zhu et al., 2015). Internodes were simulated as cylinder objects, while leaves were composed of 100 individual parallelograms that varied in width along the leaf according to (Equation 2) with cultivar-specific or fixed parameter values. Expansion of internodes and leaves was calculated using the sigmoid function y(t) = ymax/1 + e (-k * (t – thalf)) with k = 0.05 and thalf = 40°Cd. Successive leaf appearance was simulated based on growing degree days (local daily minimum and maximum temperature, Tbase = 8°C) and a phyllochron of 30°Cd. The angle between consecutive leaves was 160°. To ensure that only differences in leaf shape were assessed in the simulations, (i) the maximum leaf length and the length-to-width ratio (LWRatio) of each leaf rank were derived from the measured data as an average of the seven cultivars, and (ii) the width was further normalized to maintain a consistent leaf area across different shapes (**Figure 4B**). Therefore, all parameters related to plant structure – such as expansion dynamics, final internode dimensions (length and radius, and leaf attributes including length, length-to-width ratio, angle, and curvature – were kept identical across all cultivars. Additionally, leaf length and length-to-width ratio were averaged per rank across cultivars to ensure consistency. Since leaf shape influences total leaf area even when length and width remain unchanged, it is impossible to analyze leaf shape in isolation. To account for this, leaf width was adjusted to achieve uniform leaf area across different shapes, following the Equations 3, 4:

(3) Leaf width (adj)=leaf length∗(1/LWRatio)∗(0.75/c)

(4) Leaf Area=Length∗Width (adj)∗c

where *c* is the leaf area coefficient, obtained by integrating the leaf shape function in 100 steps from 0.01 to 1.0. This coefficient defines the percentage of a rectangle of a given width and length that is occupied by the leaf. The commonly used factor of *c* = 0.75 (Sanderson et al., 1981) was applied for normalization. Consequently, leaf width increases for *c* < 0.75 and decreases for *c* > 0.75. Ultimately, regardless of leaf shape, leaf area remained constant. Simulations were run from the date of sowing until the last measurement date for a total of 81 days. The plant canopy was designed equivalent to the field experiment with a row distance of 0.75 m and a plant density of 9 plants m^-2^, resulting in a plant distance within the row of 0.15 m. Six rows with 14 plants each were simulated. The architecture of all plants in a simulation was uniform, regardless of their position within the canopy and the amount of light they individually intercepted. Daily light interception per leaf rank was averaged for the plants in the centre of the simulated scene to avoid border effects. To evaluate differences in canopy light interception between cultivars, the sum of all leaf ranks was taken, and the value per cultivar was divided by the maximum value of all cultivars. Each simulation was run twice to estimate the standard errors, which were always small due to the large number of light rays and the high reflection depth.

To further investigate extreme leaf shape characteristics, different combinations of term *alpha* and term *a* ranging from 0.5 to 1.0 were analyzed. Low values resulted in more elliptical leaves while high values resulted in more lanceolate leaves. Throughout these simulations, leaf area was kept constant to ensure that observed changes resulted solely from differences in leaf shape. This approach quantified the maximum effect on light interception outside the range of the leaf shapes found for the cultivars in this study. The statistical analysis was performed using multiple linear regression in SAS 9.4 (SAS Institute Inc., 2016). The model included the term *alpha* and term *a* as well as their interactions in order to evaluate their influence on light interception. The significance of the main effects and interactions was determined using F-tests from the regression analysis.

### 2.5 Statistical analysis between cultivars and leaf ranks

To detect significant differences between cultivars and leaf ranks in the estimated parameters of term *alpha* and term *a*, a mixed model was fitted with SAS 9.4 (SAS Institute Inc., 2016) according to Equation 5:


(5) $$y_{jklmn}=\mu +b_{l}+r_{m}+p_{lmn}+\tau_{j}+\varphi_{k}+{\left(\tau \varphi \right)}_{jk}+\;e_{jklmn}$$

where $y_{jklmn}$ is the parameter estimate from leaf rank *k* of plant *n* grown in the *l*-th bock and *m*-th row of cultivar *j*, μ is the intercept, $b_{l}$ , $r_{m}$ , and $p_{lmn}$ are the random effects of block *l*, row *m* and plant *n* grown in block *l* and row *m*. The terms $\tau_{j}$ , $\varphi_{k}$ , and ${\left(\tau \varphi \right)}_{jk}$ are the fixed treatment effects of cultivar *j* and leaf rank *k* and their interaction effects, and $e_{jklmn}$ is the error of $y_{jklmn}$ . Block and row were included as random effects to account for spatial variability, while plants nested within row and block controlled for individual variability in leaf morphology, showing improved model accuracy. Error effects of the same plant were assumed to have a common variance-covariance structure. A first-order autoregressive plus nugget variance-covariance structure was assumed with leaf rank-specific variance. The variance of errors of the first-order autoregressive part was assumed to be proportional to the variance of the estimates, as a weighted analysis was performed. Normal distribution and homogeneous variance of residuals were checked graphically for both parameters via residual plots. Least-squares (LS) means were calculated and a Tukey-Test (α = 0.05) was performed. Multiple mean comparisons were then presented via letter display.

## 3 Results

### 3.1 Evaluation of model fit for estimated relative width

**Figure 5:**
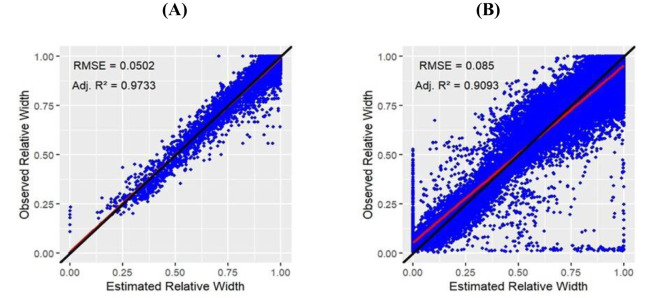
Linear regression analysis between estimated relative width and observed relative width data in (Equation 2) of manual method (I) **(A)** and camera method (II) **B)**.

The evaluation of data obtained using the manual method (I) was based on 4654 data points, whereas the camera method (II) provided a much higher data density of 68586 data points. Linear regression analysis evaluating the relationship between estimated relative width and observed relative width resulted in adj. R² = 0.9733 and RMSE = 0.0502 for the manual method (I) (**Figure 5A**) and adj. R² = 0.9093 with RMSE = 0.0850 for the camera method (II) (**Figure 5B** **).**

Function fits were applied to data from both methods (I) and (II), allowing leaf shapes to be determined for all measured specimens. In some cases, the contour detection of the camera method (II) produced outliers, reflecting the relationship between high data density and software resolution. Notably, all recorded individual images were used for data generation and processing using a single evaluation algorithm. Detailed individual fit results for each measurement and method are provided (**Supplementary Figures 3-8**).

#### 3.1.1 Regression and correlation analysis of LS means for term *alpha* and term *a*

Both methods were compared with regard to the term alpha and term a in order to check the reproducibility and accuracy. For term *alpha*, regression analysis on the observed relative leaf width yielded an adj. R² = 0.7513 and RMSE = 0.1414. For term *a*, regression analysis resulted in adj. R² = 0.4591 and RMSE = 0.0377.

### 3.2 Comparative statistical analysis of cultivars and leaf ranks

Statistical analysis comparing cultivars and leaf ranks revealed significant differences (p < 0.01) for term *alpha* and term *a* according to (Equation 2). These differences were observed for the fixed effects of cultivar, rank, and their interactions. The results obtained from the manual method (I) are provided (**Supplementary Figures 9, 10**). As the primary focus of this study is the newly developed camera-based method (II), its results will be presented in the following.

### 3.3 Camera method (II): term *alpha*

#### 3.3.1 Rank-by-cultivar differences

**Figure 6:**
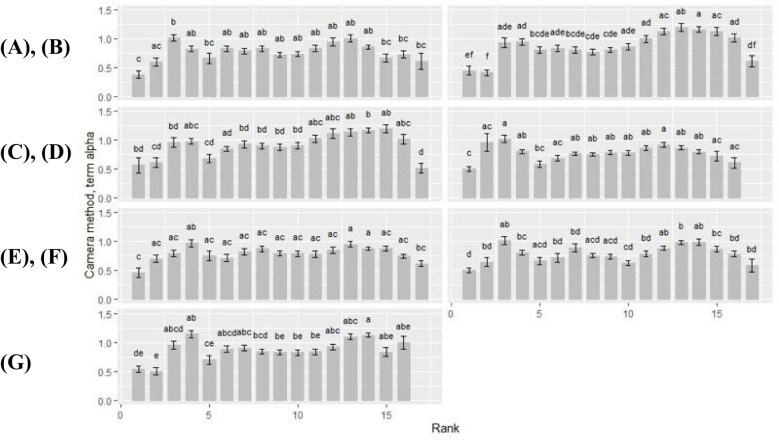
Camera method (II) data for term *alpha*. Rank-by-cultivar differences were analyzed for **(A)** Amaroc; **(B)** Benedictio; **(C)** Figaro; **(D)** LG30.258; **(E)** Ricardinio; **(F)** Ronaldinio; **(G)** Stabil. Results are presented as least square mean ± standard error (LSmean ± SE). Means with at least one identical letter are not significantly different from each other as indicated by the Tukey-Test (α = 0.05).

Overall, term *alpha*, representing the ‘leaf shape’ factor, showed a wavy curve profile for all rank-by-cultivar differences, with peak values around rank 4 and rank 14 (**Figure 6**). At first rank, term *alpha* became the lowest (*alpha* ≤ 0.56), which was observed independent of the cultivar. Therewith, the term *alpha* always became significantly lower compared to the other plant ranks. For the cultivars Benedictio and Stabil, rank 2 was also below this limit. An exception was found for the cultivar Figaro, with its minimum at rank 17 for the term *alpha* (*alpha* = 0.51). In contrast, maximum values for *alpha* varied depending on the cultivar representing shape differences. For cultivar Amaroc, a maximum across all ranks was found at rank 3 (*alpha* = 1.01), and for cultivar LG30.258 (*alpha* = 1.02) and Ronaldinio (*alpha* = 1.02). At rank 4, however, the term *alpha* showed a maximum value for cuextreltivar Ricardinio (*alpha* = 0.97) as well as for cultivar Stabil (*alpha* = 1.15). For cultivar Benedictio, the maximum was found at rank 13 (*alpha* = 1.20), and for Figaro, it was found at rank 15 (*alpha* = 1.19).

#### 3.3.2 Cultivar-by-rank differences

Cultivar-by-rank differences became significant at rank 4, 14 and 15 (**Table 2**). At rank 4, the term *alpha* of cultivar LG30.258 (*alpha* = 0.80) and Ronaldinio (*alpha* = 0.81) was significantly lower compared to cultivar Stabil (*alpha* = 1.15). At rank 14, cultivar Amaroc (*alpha* = 0.86), LG30.258 (*alpha* = 0.80), and Ronaldinho (*alpha* = 0.99) were significantly different from cultivar Benedictio (*alpha* = 1.16), Figaro (*alpha* = 1.16), and Stabil (*alpha* = 1.13). At rank 15, cultivar Amaroc (*alpha* = 0.67) was significantly lower compared to cultivar Benedictio (*alpha* = 1.13) and Figaro (*alpha* = 1.19).

**Table 2:** Camera method (II) for term *alpha*. Significant cultivar-by-rank differences at rank 4, 14 and 15.

Cultivar-by-rank			Cultivar	LSmean	HSD (α = 0.05)
Cultivar	rank	4	Amaroc	0.83 ab	0.24
			Benedictio	0.95 ab	
			Figaro	0.98 ab	
			LG30.258	0.80 b	
			Ricardinio	0.97 ab	
			Ronaldinio	0.81 b	
			Stabil	1.15 a	
Cultivar	rank	14	Amaroc	0.86 b	0.19
			Benedictio	1.16 a	
			Figaro	1.16 a	
			LG30.258	0.80 b	
			Ricardinio	0.87 b	
			Ronaldinio	0.99 ab	
			Stabil	1.13 a	
Cultivar	rank	15	Amaroc	0.67 b	0.31
			Benedictio	1.13 a	
			Figaro	1.19 a	
			LG30.258	0.72 ab	
			Ricardinio	0.88 ab	
			Ronaldinio	0.86 ab	
			Stabil	0.84 ab	

Results are presented as least square mean ± Tukey’s Honestly Significant Difference (HSD) at *α* = 0.05 (LSmean ± HSD). Means with at least one identical letter are not significant different from each other as indicated by Tukey-Test (α = 0.05).

### 3.4 Camera method (II): term *a*

#### 3.4.1 Rank-by-cultivar differences

**Figure 7:**
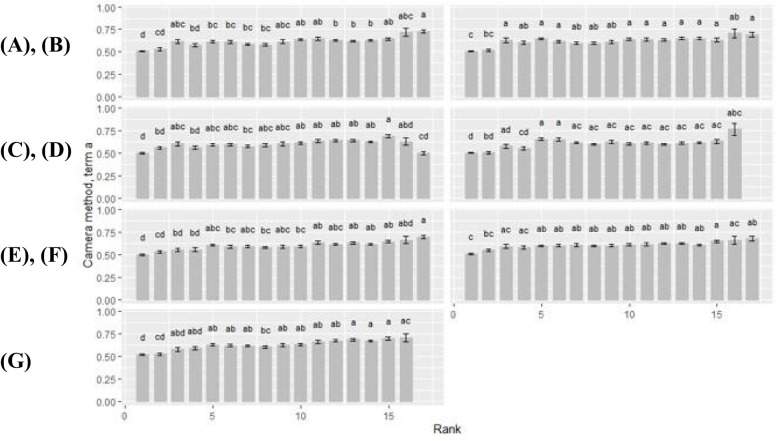
Camera method (II) data for term *a*. Rank-by-cultivar differences were analyzed for **(A)** Amaroc; **(B)** Benedictio; **(C)** Figaro; **(D)** LG30.258; **(E)** Ricardinio; **(F)** Ronaldinio; **(G)** Stabil. Results are presented as least square mean ± standard error (LSmean ± SE). Means with at least one identical letter are not significantly different from each other as indicated by the Tukey-Test (α = 0.05).

For all cultivars, term *a* increased with rank (**Figure 7**). Within each cultivar, term *a* was minimal at rank 1 with *a* ≤ 0.52. Exceptions were found at rank 2 of cultivar Benedictio and LG30.258, and at rank 17, cultivar Figaro, where term *a* was below this limit, too. Maximum values ranged from 0.63 to 0.77 at upper ranks (rank >15) across all cultivars. For cultivar Figaro, the maximum for term *a* was found at rank 15 (*a* = 0.69), for cultivar Benedictio at rank 16 (*a* = 0.71) as well as for LG30.258 (*a* = 0.77) and Stabil (*a* = 0.71). For cultivar Amaroc (*a* = 0.73), Ricardinio (*a* = 0.70), and Ronaldinio (a = 0.68), the maximum was at rank 17.

#### 3.4.2 Cultivar-by-rank differences

Cultivar-by-rank interactions for term *a* showed significant differences at ranks 14 and 17 (**Table 3**). At rank 14, cultivar Stabil was significantly higher (*a* = 0.67) than the cultivars LG30.258, Ricardinio, and Ronaldinio with *a* ≤ 0.62. At rank 17, cultivar Figaro was significantly smaller with *a* = 0.50 compared to the other cultivars.

**Table 3:** Camera method (II) for term *a.* Significant cultivar-by-rank differences at rank 14 and 17.

Cultivar-by-rank			Cultivar	LSmean	HSD (α = 0.05)
Cultivar	rank	14	Amaroc	0.63 ab	0.04
			Benedictio	0.65 ab	
			Figaro	0.63 ab	
			LG30.258	0.62 b	
			Ricardinio	0.62 b	
			Ronaldinio	0.61 b	
			Stabil	0.67 a	
Cultivar	rank	17	Amaroc	0.73 a	0.30
			Benedictio	0.69 a	
			Figaro	0.50 b	
			Ricardinio	0.70 a	
			Ronaldinio	0.68 a	

Results are presented as least square mean ± Tukey’s Honestly Significant Difference (HSD) at *α* = 0.05 (LSMean ± HSD). Means with at least one identical letter are not significantly different from each other as indicated by the Tukey-Test (α = 0.05).

### 3.5 Effect of leaf shape on light interception

**Figure 8:**
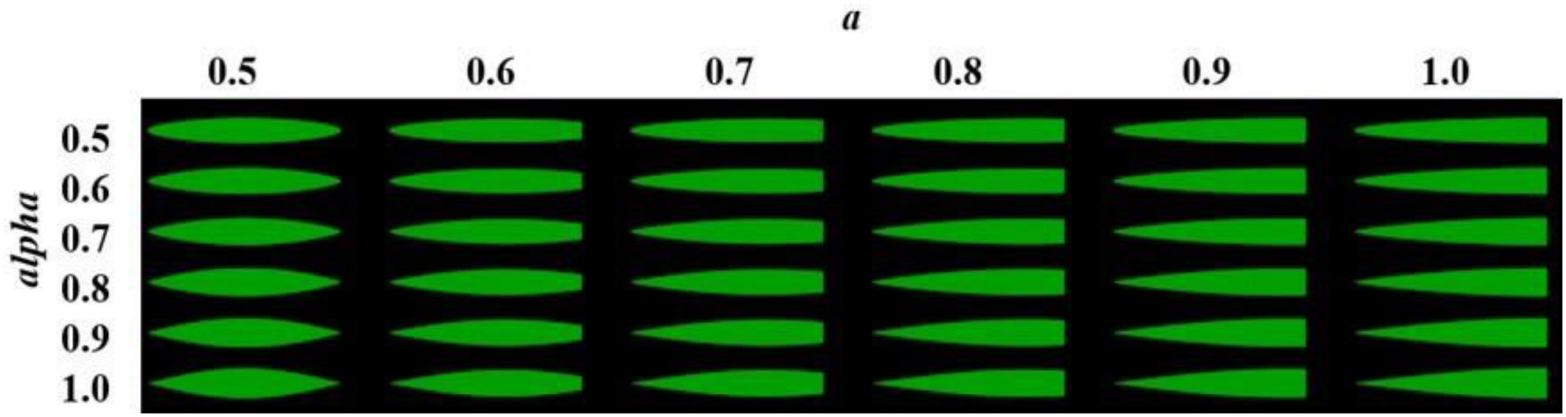
Visualization of leaf shapes calculated according to (Equation 2) with the combinations of term *alpha* and *a* between 0.5 and 1.0 in steps of 0.1.

Within the FSPM, the light interception of the entire canopy was simulated, with leaf length and leaf area set as constants to isolate the effects of leaf shape: This ensures that the observed differences stem solely from shape variations rather than other factors such as leaf size or biomass distribution. Using the previously described FSPM, maximum light interception occurred at the ranks 10 to 12, depending on the cultivar. Throughout the vegetation period, no significant differences were observed across cultivars. The increase in light interception between 30 and 60 days after sowing followed a similar trajectory for all cultivars. Differences became evident only at the end of the simulation (**Table 4**). To assess rank-dependent effects, rank 14 was analyzed separately after term *alpha* and term *a* were identified as significant for the interaction between cultivar and rank. Canopy-level light interception ranged from 0.98 (cultivars Amaroc and Ronaldinio) to 1.00 (cultivar Benedictio). At rank 14, greater variability was observed between cultivars, ranging from 0.93 (cultivar Ricardinio) to 1.00 (cultivar Stabil). Since no statistically significant differences in canopy-level light interception were detected among the seven maize cultivars, further simulations were conducted to examine how extreme leaf shapes would influence light interception (**Figure 8**). As a result of these more extreme leaf shapes, light interception declined by up to 7%, depending on the term *alpha* and term *a* (**Table 5**). Statistical analysis showed a significant effect of term *alpha* on light interception (p = 0.0098) and a significant interaction effect (p = 0.0016). However, term *a* was not found to be significant (p = 0.1670).

**Table 4:** Simulated canopy light interception relative to the maximum for the seven cultivars at the end of the simulation at 81 days after sowing.

Cultivar	Relative light interception	
	Canopy	Rank 14
Amaroc	0.98	0.97
Benedictio	1.00	0.96
Figaro	0.99	0.94
LG30258	0.99	0.99
Ricardinio	0.99	0.93
Ronaldinio	0.98	0.97
Stabil	0.99	1.00

**Table 5:** Simulated canopy light interception (relative to maximum), 81 days after sowing for different leaf shapes calculated according to (Equation 2) with the combinations of term *alpha* and term *a* between 0.5 and 1.0 in steps of 0.1.

*alpha*	*a*					
	0.50	0.60	0.70	0.80	0.90	1.00
0.50	0.97	0.97	0.97	0.98	0.96	0.96
0.60	0.99	0.98	0.97	0.97	0.95	0.96
0.70	0.99	0.98	0.96	0.97	0.96	0.95
0.80	1.00	0.98	0.96	0.96	0.96	0.96
0.90	1.00	0.98	0.95	0.95	0.94	0.94
1.00	1.00	0.96	0.95	0.96	0.94	0.93

## 4 Discussion

The camera method (II) was developed as an computer vision-based approach for determining the maximum leaf width of destructively sampled leaves along the leaf blade using computer vision within a controlled standard indoor measurement environment. Validation of the camera method (II) was based on pixel counting and green-non-green contrast analysis, which were converted into metric measurements and compared with manual method (I) data. With the automatic evaluation algorithm, thousands of images could be analyzed which offered a time-efficient and high throughput rate. However, it should be noted that leaf overlaps or curvatures can affect contour detection, resulting in slight distortions in shape detection. Therefore, a controlled lighting environment with minimal dust exposure is recommended to maximize measurement accuracy. This study analyzed images from video recordings of maize leaves using a single software configuration. However, the software is fully customizable, allowing precise adjustments based on user-defined image processing parameters. Refining these parameters could further improve the accuracy of contour detection. Such adjustments would be especially beneficial for smaller datasets, where individual image evaluations can be manually refined. Finally, the measured width for each segmented leaf was stored in an output file. Compiling these output files generated a comprehensive dataset of observed relative leaf width data along the blade for the camera method (II). Fitting (Equation 2) to the data allowed for estimated leaf width values and leaf shape parameters by rank and cultivar, demonstrating both feasibility and reproducibility.

To preselect the appropriate leaf shape function and to validate the camera method, the regression analysis was first performed on the data collected using the manual method (I). The regression fit demonstrated that the equation according to Bos et al. (2000) provided the best overall model fit, yielding adj. R² = 0.9871 and RMSE = 0.0349, compared to (Equation 2), which resulted in adj. R² = 0.9733 and RMSE = 0.0502. Moreover, with a sample size of 450 leaves, the term *beta* significantly deviated from 1 (p = 0.0273; *beta* = 0.89; 95% confidence interval: 0.788 to 0.985). However, the difference from (Equation 2) was considered negligible for the final estimation. In this regard, note that adj. R² was consistently used to account for the number of estimated parameters. The correlation between the estimated relative width values using (Equation 2) and the equation according to Bos et al. (2000) yielded a Pearson correlation coefficient of 0.9924, indicating that both models resulted in highly correlated estimates of relative width It is important to note that this study focused on treatment differences; therefore, possible scale-level variation in (Equation 2) and the alternative equation of Bos et al. (2000) were irrelevant. (Equation 2), according to Sanderson et al. (1981), was developed specifically for estimating leaf width in plant growth models.

A fundamental challenge in plant modeling is to achieve an optimal balance between complexity and variability. Models must be sufficiently detailed to simulate biological mechanisms accurately. However, increasing complexity can compromise stability and increase the risk of errors and uncertainties. Consequently, overparameterization of FSPMs should be minimized to improve computational efficiency through model simplification and optimization of functional performance (Chapagain et al., 2022; Pasley et al., 2022). Regression analysis confirmed the high accuracy of function fitting, validating both the feasibility and reliability of the new camera method (II). Occasional outliers likely resulted from minor distortions due to the uniform image evaluation algorithm. Statistical analysis of LS means by rank and cultivar revealed significantly lower values for term *alpha* at rank 1 across all cultivars, reinforcing the correlation between leaf shape and rank position (Bos et al., 2000). Compared to the manual method (I), the camera method (II) produced a smoother rank profile, likely due to its tenfold increase in measurement points and contour detection algorithm.

Cultivar-specific differences in maximum values of term *alpha* indicated significant rank-by-cultivar interactions. These findings support the validity of the camera method (II) in capturing leaf shape variations with high consistency. Among the seven maize cultivars tested, distinct cultivar-by-rank variations were observed for term *alpha* at rank 4, 14, and 15 (Bos et al., 2000; Sanderson et al., 1981). Likewise, for the ‘ratio factor’ term *a*, a rank-dependent increase was observed toward the final ranks across all cultivars except for cultivar Figaro, with significant differences at ranks 14 and 17. These results align with Dornbusch et al. (2011), who reported rank-related leaf shape variations across different cultivars, independent of sowing date and growth conditions, while emphasizing that cultivar-specific discrepancies remained minimal. For the FSPM, estimated parameters for term *alpha* and term *a* were obtained using the camera method (II). The simulations revealed minor variations in canopy-level light interception among the seven maize cultivars evaluated. At 81 days after sowing, light interception values remained high across most ranks (0.98 to 1.00); however, rank 14 exhibited significant differences, ranging from 0.93 to 1.00. These subtle variations may originate from genetic differences between cultivars, further confirming the suitability of FSPM for analyzing feedback mechanisms between physiological processes – both at the level of individual plant organs and at the canopy level (Vos et al., 2010). To enhance the applicability of these findings, future studies should examine maize cultivars of different maturity groups to enable a more comprehensive characterization of light interception dynamics. Additionally, incorporating data from multiple locations and growing seasons would account for environmental variability, further improving model accuracy and predictive capacity (Dias et al., 2019).

Although the estimated leaf shapes of the seven maize cultivars evaluated in this study did not significantly impact light interception at the canopy level, simulations of more extreme leaf shapes revealed variations of up to 7%. These findings underscore the importance of precise leaf parameterization and highlight that leaf shape variability can substantially impact light interception. Furthermore, the most extreme configuration (term *alpha* = 1; term *a* = 1.0) exhibited maximum leaf width at the base, potentially leading to increased shading effects and altered light distribution within the canopy. In detail, our results demonstrate that, that the term *alpha* as the ‘shape factor’ had a significant effect on light interception, whereas the term *a* as the ‘ration factor’ did not show a statistically significant effect. The strong interactions between these terms underscores the need to consider interaction effects when modeling light interception. Our findings align with previous studies (Bos et al., 2000; Dornbusch et al., 2011; Sanderson et al., 1981), suggesting that a more detailed leaf parameterization may be necessary to simulate different cultivars correctly. It is evident that leaf shape, in combination with leaf orientation, shading dynamics, and the overall structure of the canopy, plays a fundamental role in determining the efficiency of light interception. Intra-canopy light distribution and shading patterns are influenced by variability in leaf shape and size, thereby modulating photosynthetic performance and potentially altering radiation use efficiency. However, leaf shape must also be part of a broader architectural framework, interacting with factors such as leaf expansion, leaf area, leaf angle, plant growth characteristics, plant density, and competition. These factors collectively regulate canopy light interception, biomass accumulation, and overall crop productivity (Collison et al., 2020; Yang et al., 2019). Further investigations are therefore needed to test to which extend leaf shape variability translates into physiological changes and affects biomass accumulation and yield under various conditions.

With regard to leaf area estimation, Sanderson et al. (1981) demonstrated that by applying (Equation 2) for leaf width considering fixed parameters (term *alpha* = 0.85; term *a* = 0.70), leaf area of fully developed leaves can be accurately calculated. The leaf area (LA) of fully developed leaves is typically determined using the formula LA=c × LL ×LW  , where *c* is a crop-specific leaf area coefficient, LW is maximum leaf width, and LL is leaf length. For maize, regardless of rank, growth stage, or cultivar, the leaf area coefficient *c* = 0.75 is most commonly used (Montgomery, 1911; Sanderson et al., 1981). However, to enhance the accuracy of leaf area estimation, this coefficient should be treated as a dynamic variable, adapting as leaf expansion and shape modifications occur during growth (Dornbusch et al., 2011; Schrader et al., 2021). For *Poaceae* species, including maize, leaf area coefficients actually range from 0.70 ≤ *c* ≤ 0.75. Our simulations indicate that rank-specific leaf shape variability allows precise leaf area coefficient estimation, utilizing relative leaf length and the leaf shape to predict term *alpha* and term *a*. The camera method (II) could also be used to estimate leaf area coefficients for non-destructive evaluation of leaf developmental rates. When term *alpha* ranged from 0.5 ≤ *alpha ≤* 1.0 in increments of 0.1 while term *a* remained constant (*a* = 0.7), integrated leaf width functions in the FSPM resulted in notable alterations in the cumulative leaf area, confirming the calculations of Sanderson et al. (1981). These results suggest that a rank-dependent leaf area coefficient could significantly improve the accuracy of leaf area estimation through scaling functions, considering the estimated leaf size, as proposed by Schrader et al. (2021). During leaf expansion, the leaf area coefficient can vary, for example, between 0.68 < *c* < 0.82 for *alpha* = 0.7.

The overall results emphasize the importance of dynamic parameterization of leaf shape to enhance the accuracy of leaf area estimation in maize. Additionally, they highlight the potential of the proposed camera method (II) for broader applications in crop modeling and phenotyping. Although the presented camera method (II) was primarily developed for maize (*Zea mays* L.) and tested on seven cultivars of the same maturity group, we see the potential that the method may be applied to other maize cultivars and maturity groups and under diverse environmental conditions. Additionally, the camera method (II) could be transferred to other crops with large, undulating leaves, for example sorghum (*Sorghum bicolor*) or sunflower (*Helianthus annuus*). However, adapting the camera method (II) to different species than maize may require modifications in image processing, particularly in segmentation and calibration, to accommodate species-specific morphological traits. Future research should systematically investigate the possibility to transfer this approach to additional plant species beyond maize, particularly regarding rank-dependent leaf shape variations. Expanding the applicability of the camera-based method (II) could improve canopy structure representation in FSPMs, leading to a more realistic depiction of plant architecture, light utilization, and biomass production.

## 5 Conclusion and outlook

This study introduced a computer vision-based method for fast and accurate measurement of maize leaf width in a controlled indoor environment, utilizing a GoPro Hero Black camera and an LI-3100C Area Meter. The method effectively captured the position of maximum leaf width, enabling precise characterization of rank-based leaf shape variations according to the equation by Sanderson et al. (1981). The Python-based software tool developed for video sequence and image analysis was applied in this study using a single configuration for all images, ensuring consistency in the evaluation process, with no individual images discarded. While the standardized setup proved effective for large datasets, an individualized configuration could further improve contour detection accuracy, particularly for smaller datasets. The software tool is publicly available as part of this publication. By integrating estimated leaf width values into a functional-structural plant model (FSPM), simulations demonstrated that extreme leaf shapes can alter light interception by up to 7%, highlighting the importance of precise parameterization. The method provides a scalable, non-destructive approach for estimating leaf area in developing maize plants and has potential applications in phenotyping. Future research should explore its adaptability to other crops with large, undulating leaves while refining segmentation and calibration techniques for species-specific traits. Additionally, incorporating leaf shape dynamics – such as expansion rates and specific leaf area – into FSPMs would enhance yield predictions and deepen our understanding of plant growth processes.

## Data Availability

The original contributions presented in the study are included in the article/**Supplementary Material**. Further inquiries can be directed to the corresponding author.
