# Supplementary material for: A computer vision approach for quantifying leaf shape of maize (Zea mays L.) and simulating its impact on light interception
Source: Front Plant Sci. 2025 Jun 23;16:1521242. doi: 10.3389/fpls.2025.1521242 (PMC12230053; doi:10.3389/fpls.2025.1521242)
Supplement: Supplementary file 1 [file DataSheet1.pdf]

Supplementary Material

Supplementary Table 1 F-Test of manual method (I)

**Effect**	**Term *alpha***	**Term *a***
	Significance p < 0.05	
**Cultivar**	<0.001	0.0029
**Rank**	<0.001	<0.0001
**Cultivar x Rank**	<0.013	<0.0001

Supplementary Table 2 F-Test of camera method (II)

**Effect**	**Term *alpha***	**Term *a***
	Significance p < 0.05	
**Cultivar**	<0.001	0.0073
**Rank**	<0.001	<0.001
**Cultivar x Rank**	<0.001	0.0043

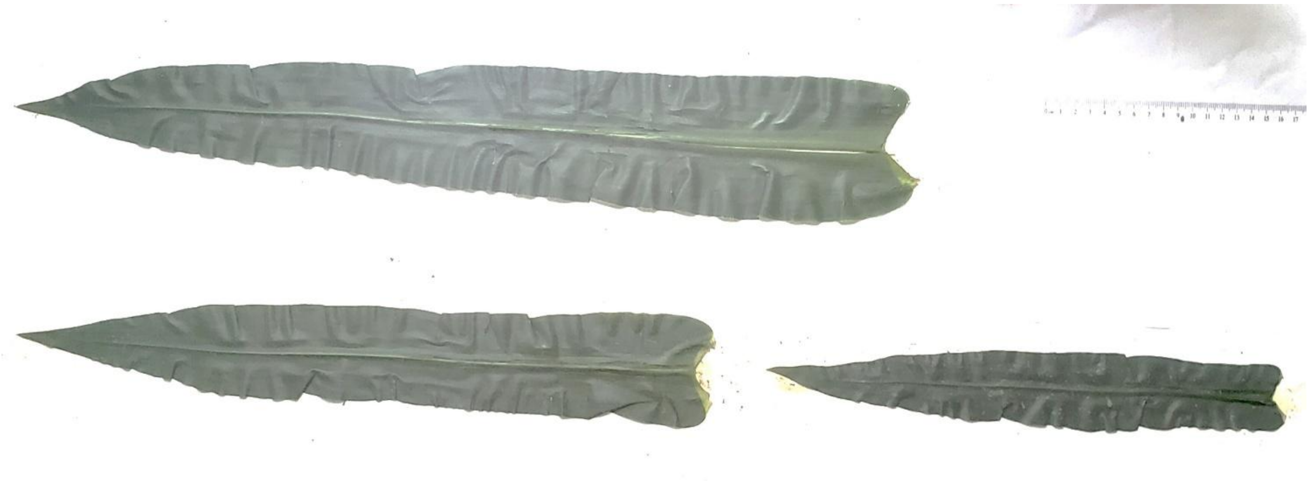

Supplementary Figure 1 Flattened maize leaves between acrylic glass

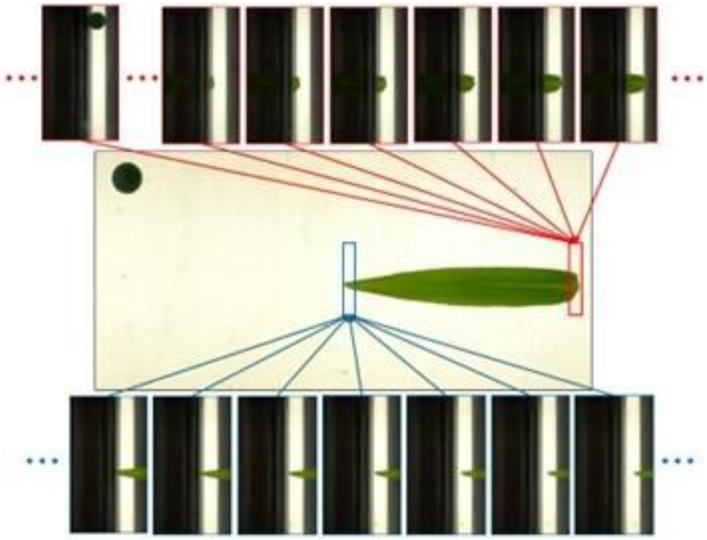

Supplementary Figure 2 Video-framing with the camera method (II)

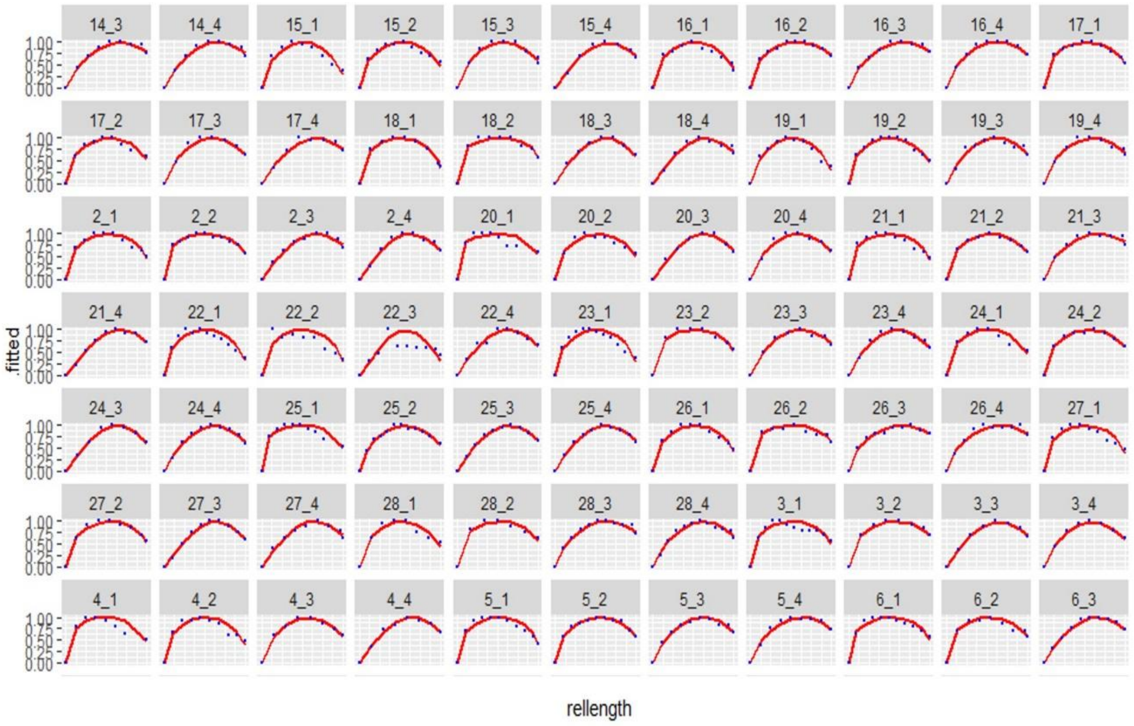

Supplementary Figure 3 Manual method (I) data in equation (3). Examples for fitting plant-rank specific data (relative length to relative width, scale 0 to 1) at M1.

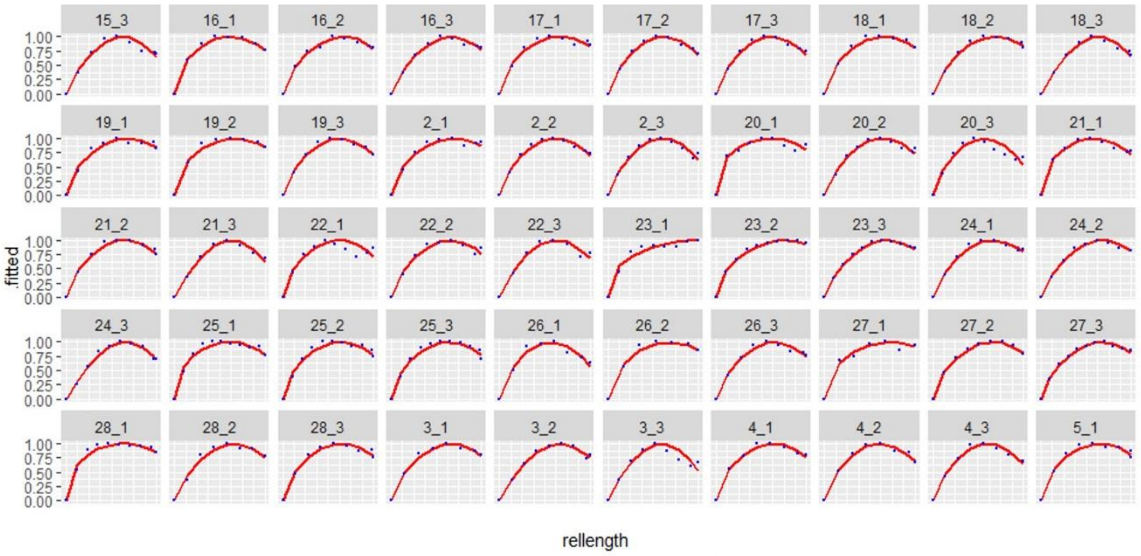

Supplementary Figure 4 Manual method (I) data in equation (3). Examples for fitting plant-rank specific data (relative length to relative width, scale 0 to 1) at M2.

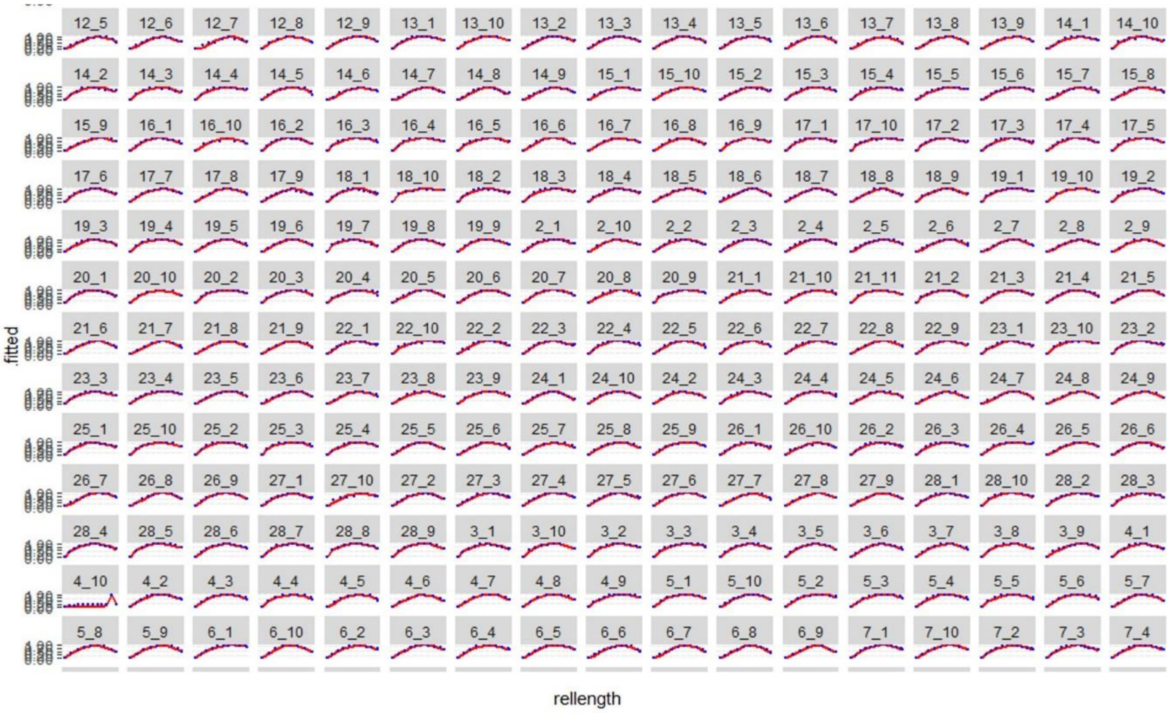

Supplementary Figure 5 Manual method (I) data in equation (3). Examples for fitting plant-rank specific data (relative length to relative width, scale 0 to 1) at M3.

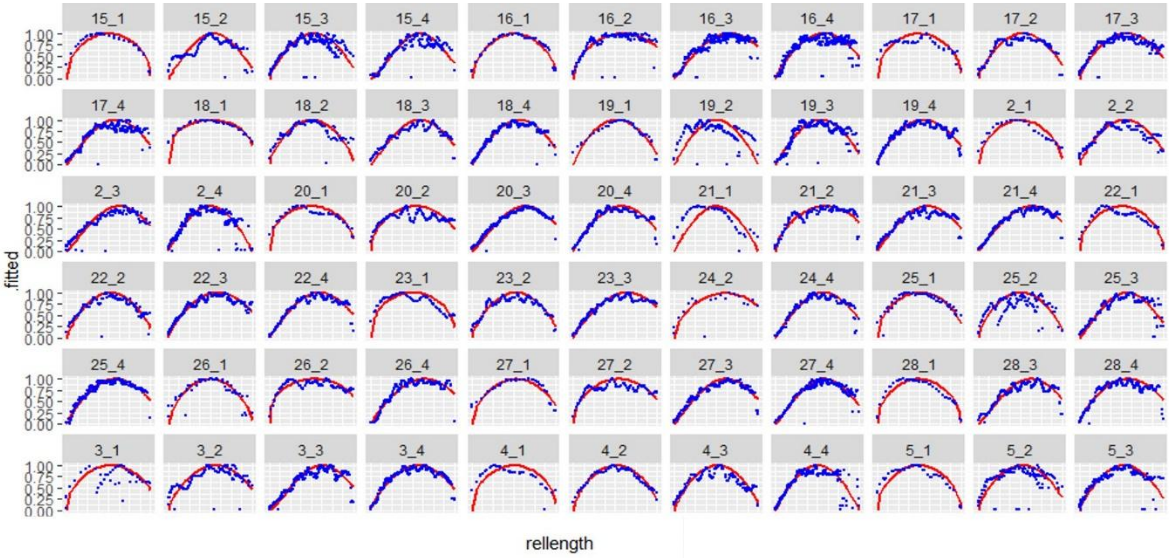

Supplementary Figure 6 Camera method (II) data in equation (3). Examples for fitting plant-rank specific data (relative length to relative width, scale 0 to 1) at M1.

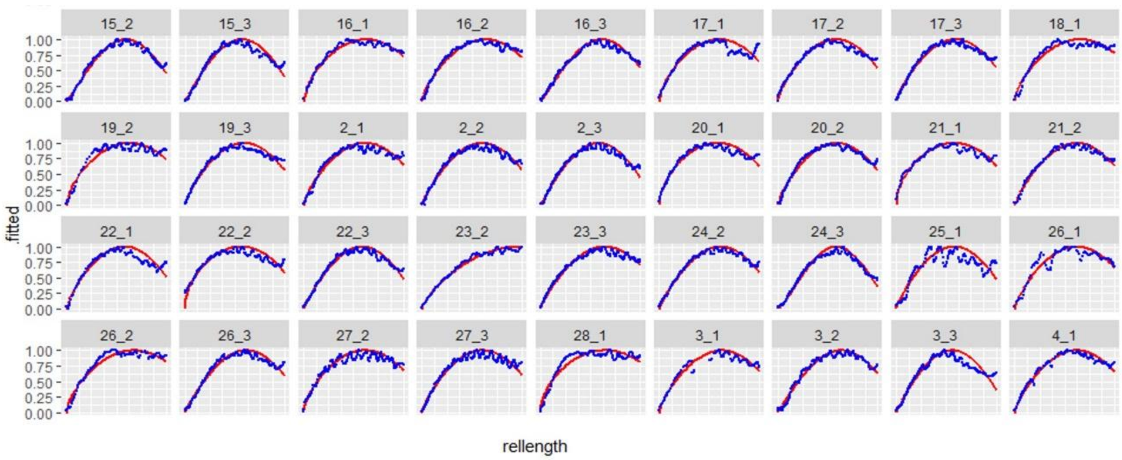

Supplementary Figure 7 Camera method (II) data in equation (3). Examples for fitting plant-rank specific data (relative length to relative width, scale 0 to 1) at M2.

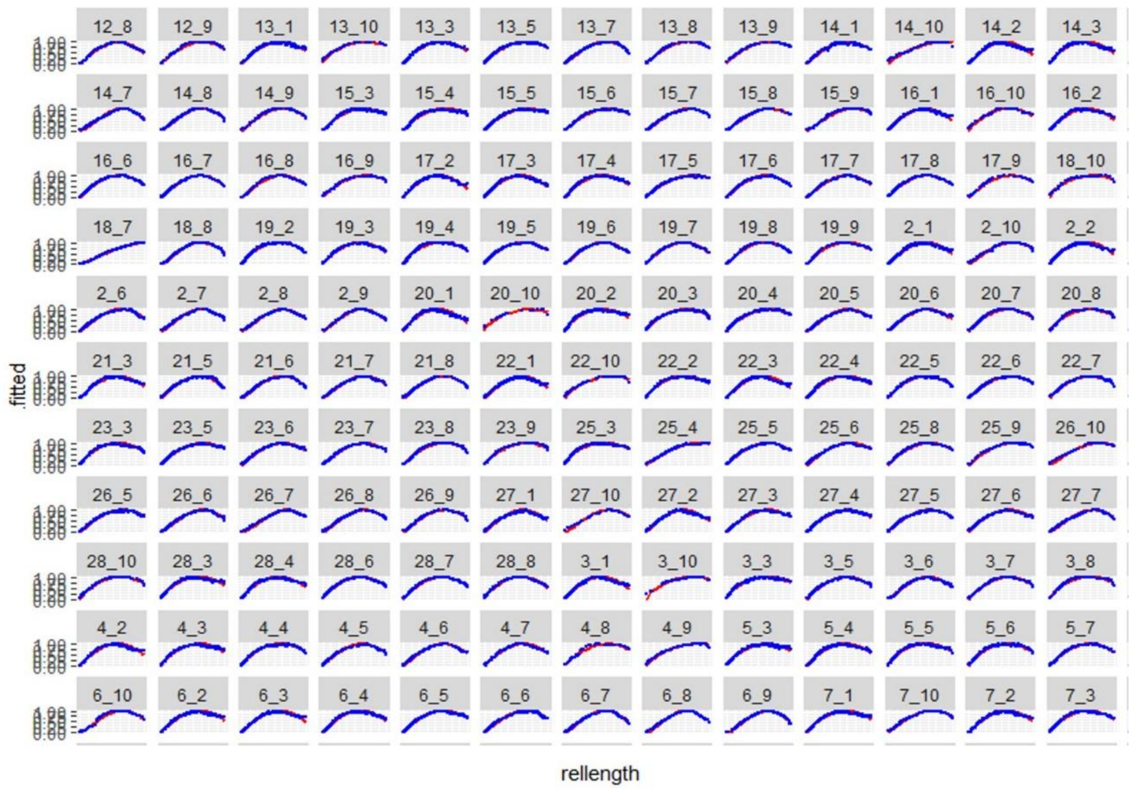

Supplementary Figure 8 Camera method (II) data in equation (2). Examples for fitting plant-rank specific data (relative length to relative width, scale 0 to 1) at M3.

(A), (B)

(C), (D)

(E), (F)

(G)

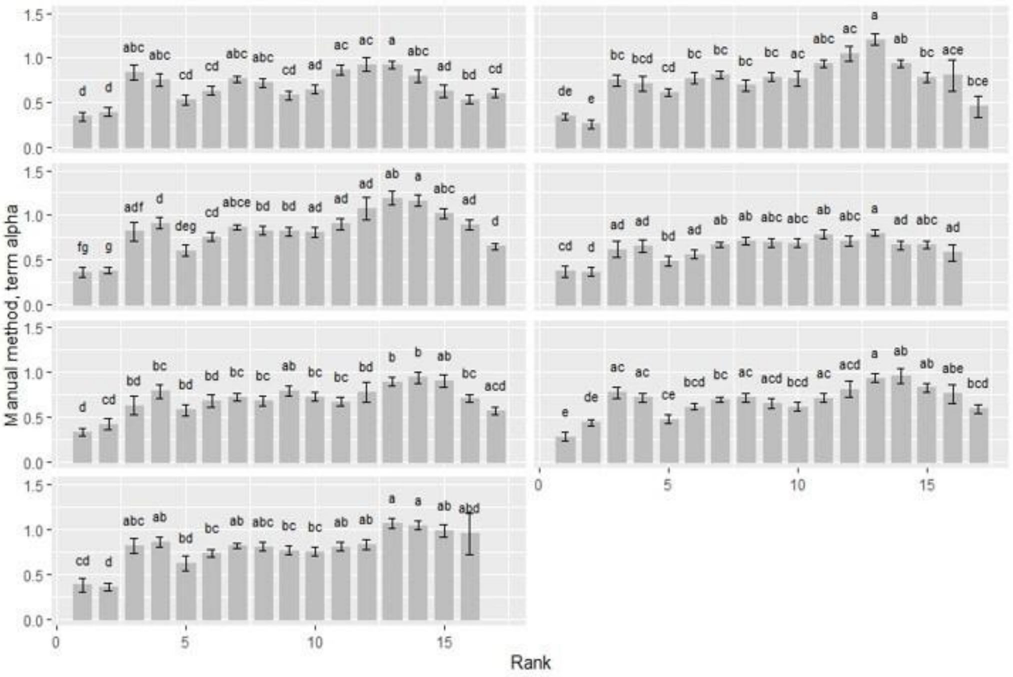

Supplementary Figure 9 Manual method (I) data for term *alpha*. Rank-by-cultivar differences were analyzed for A = Amaroc; B = Benedictio; C = Figaro; D = LG30.258; E = Ricardinio; F = Ronaldinio; G = Stabil. Results are presented as least square mean ± standard error (LSmean ± SE). Means with at least one identical letter are not significantly different from each other as indicated by the Tukey-Test ($\alpha = 0.05$).

(A), (B)

(C), (D)

(E), (F)

(G)

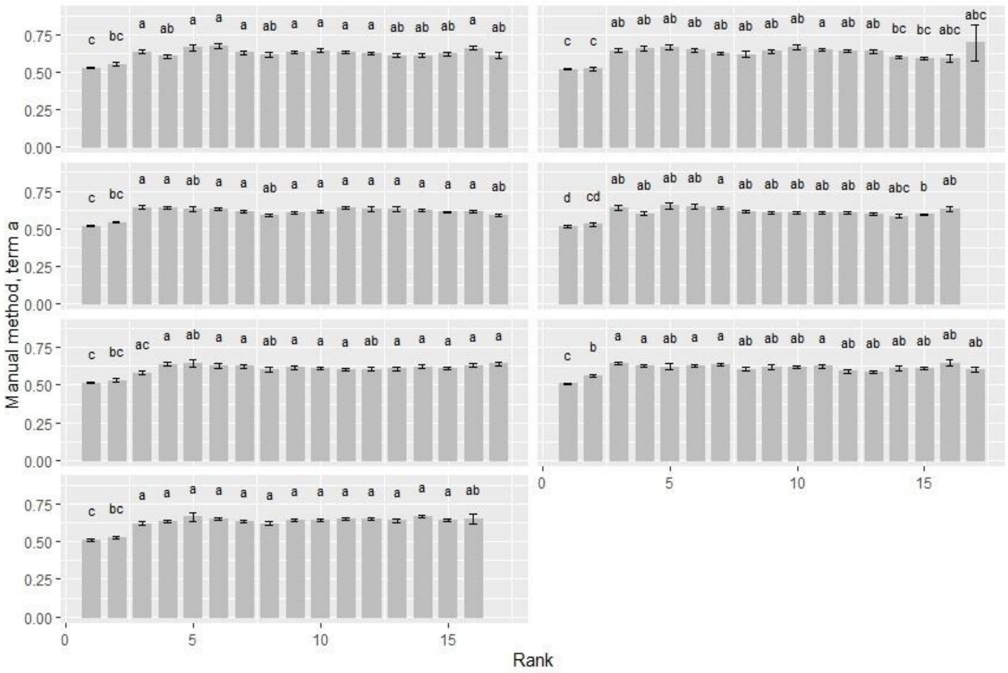

Supplementary Figure 10 Manual method (I) data for term *a*. Rank-by-cultivar differences were analyzed for A = Amaroc; B = Benedictio; C = Figaro; D = LG30.258; E = Ricardinio; F = Ronaldinio; G = Stabil. Results are presented as least square mean ± standard error (LSmean ± SE). Means with at least one identical letter are not significantly different from each other as indicated by the Tukey-Test (α = 0.05).
